# High Rate of Subclinical Chikungunya Virus Infection and Association of Neutralizing Antibody with Protection in a Prospective Cohort in The Philippines

**DOI:** 10.1371/journal.pntd.0003764

**Published:** 2015-05-07

**Authors:** In-Kyu Yoon, Maria Theresa Alera, Catherine B. Lago, Ilya A. Tac-An, Daisy Villa, Stefan Fernandez, Butsaya Thaisomboonsuk, Chonticha Klungthong, Jens W. Levy, John Mark Velasco, Vito G. Roque, Henrik Salje, Louis R. Macareo, Laura L. Hermann, Ananda Nisalak, Anon Srikiatkhachorn

**Affiliations:** 1 Department of Virology, Armed Forces Research Institute of Medical Sciences, Bangkok, Thailand; 2 Philippines-AFRIMS Virology Research Unit, Cebu City, Philippines; 3 Cebu City Health Department, Cebu City, Philippines; 4 National Epidemiology Center, Department of Health, Manila, Philippines; 5 Department of Epidemiology, Johns Hopkins School of Public Health, Baltimore, Maryland, United States of America; 6 Department of Medicine, University of Toronto, Toronto, Ontario, Canada; 7 Division of Infectious Diseases and Immunology, Department of Medicine, University of Massachusetts Medical School, Worcester, Massachusetts, United States of America; Centers for Disease Control and Prevention, UNITED STATES

## Abstract

**Background:**

Chikungunya virus (CHIKV) is a globally re-emerging arbovirus for which previous studies have indicated the majority of infections result in symptomatic febrile illness. We sought to characterize the proportion of subclinical and symptomatic CHIKV infections in a prospective cohort study in a country with known CHIKV circulation.

**Methods/Findings:**

A prospective longitudinal cohort of subjects ≥6 months old underwent community-based active surveillance for acute febrile illness in Cebu City, Philippines from 2012-13. Subjects with fever history were clinically evaluated at acute, 2, 5, and 8 day visits, and at a 3-week convalescent visit. Blood was collected at the acute and 3-week convalescent visits. Symptomatic CHIKV infections were identified by positive CHIKV PCR in acute blood samples and/or CHIKV IgM/IgG ELISA seroconversion in paired acute/convalescent samples. Enrollment and 12-month blood samples underwent plaque reduction neutralization test (PRNT) using CHIKV attenuated strain 181/clone25. Subclinical CHIKV infections were identified by ≥8-fold rise from a baseline enrollment PRNT titer <10 without symptomatic infection detected during the intervening surveillance period. Selected CHIKV PCR-positive samples underwent viral isolation and envelope protein-1 gene sequencing. Of 853 subjects who completed all study procedures at 12 months, 19 symptomatic infections (2.19 per 100 person-years) and 87 subclinical infections (10.03 per 100 person-years) occurred. The ratio of subclinical-to-symptomatic infections was 4.6:1 varying with age from 2:1 in 6 month-5 year olds to 12:1 in those >50 years old. Baseline CHIKV PRNT titer ≥10 was associated with 100% (95%CI: 46.1, 100.0) protection from symptomatic CHIKV infection. Phylogenetic analysis demonstrated Asian genotype closely related to strains from Asia and the Caribbean.

**Conclusions:**

Subclinical infections accounted for a majority of total CHIKV infections. A positive baseline CHIKV PRNT titer was associated with protection from symptomatic CHIKV infection. These findings have implications for assessing disease burden, understanding virus transmission, and supporting vaccine development.

## Author Summary

Chikungunya virus (CHIKV) is a re-emerging mosquito-borne pathogen for which the majority of infections have been considered to result in febrile illness. We sought to characterize the proportion of subclinical and symptomatic CHIKV infections in a prospective cohort of subjects ≥6 months old who underwent active surveillance for acute febrile illness from 2012–13 in Cebu City, Philippines. Symptomatic CHIKV infections were detected by PCR and/or ELISA in acute/convalescent blood samples. Subclinical infections were identified by neutralizing antibody seroconversion between enrollment and 12-month visits without symptomatic infection. Among 853 subjects who completed all study activities at 12 months, 19 symptomatic and 87 subclinical infections occurred (2.19 and 10.03 per 100 person-years, respectively). A positive baseline CHIKV PRNT titer was associated with 100% (95%CI: 46.1, 100.0) protection from symptomatic infection. Phylogenetic analysis showed Asian genotype closely related to strains from the recent Caribbean epidemic. These findings can help to assess disease burden, understand virus transmission, and support vaccine development.

## Introduction

Chikungunya virus (CHIKV) is a re-emerging mosquito-borne pathogen that has rapidly expanded its geographic reach over the past decade in Africa, Asia, the Indian and Pacific Ocean regions, and Europe. In December 2013, the first case of autochthonous CHIKV infection was confirmed in the Americas on the Caribbean island of Saint Martin and quickly spread to other Caribbean islands and parts of Central/South America [1,2]. The continental United States confirmed its first locally acquired case in Florida in July 2014 [3]. Given the large number of travelers to currently affected areas and the widespread distribution of appropriate *Aedes* mosquito vectors, spread to other parts of the Americas and Europe is likely [2,4].

CHIKV is a 12kb single-stranded, positive-sense RNA virus in the genus *Alphavirus* and family *Togaviridae*. CHIKV was first isolated from a patient in Tanzania during a febrile illness outbreak in 1952–53 [5,6]. The primary vectors in large human outbreaks are *Aedes aegypti* and *Aedes albopictus* with humans serving as the amplifying host in urban settings. Three CHIKV genotypes are known to circulate: West African, East/Central/South African (ECSA), and Asian. In 2005, CHIKV re-emerged in widespread epidemics in the Indian Ocean region and subsequently in Asia, perhaps aided by an A226V mutation in the envelope protein-1 (E1) gene of the ECSA genotype allowing the virus to spread more efficiently in *Ae*. *albopictus* [7]. In the current Caribbean epidemic, the circulating CHIKV has been identified as Asian genotype closely related to strains from China (2012), the Philippines (2012), and Micronesia/Yap (2013) [8]. Although *Ae*. *aegypti* is thought to be the main vector in the Caribbean outbreak, both *Ae*. *aegypti* and *Ae*. *albopictus* populations from different regions of the Americas may be able to transmit all three CHIKV genotypes [9].

The classic clinical presentation of chikungunya consists of febrile illness with symmetric polyarthralgia in the extremities [10]. In a minority of patients, arthralgias/arthritis can persist for months or even years. Severe or atypical disease is more likely in neonates, older adults and those with chronic medical conditions. Previous studies have demonstrated that a majority of infections present with acute fever accompanied by other overt symptoms. However, assumptions about the clinical presentation of CHIKV infection have largely been based on sentinel surveillance or cross-sectional serosurveys [11,12,13,14].

At present, no licensed chikungunya vaccine exists although several are in development [15,16]. Efficacy trials of vaccine candidates have been hampered by the unpredictability of chikungunya incidence in the field [15,17]. Development efforts would be enhanced by a better understanding of infection risk in endemic areas and the establishment of immune correlates of risk and protection.

Here, we present results from a prospective longitudinal seroepidemiological cohort study of acute febrile illness conducted in Cebu City, Philippines from 2012–13. Chikungunya was first reported in the Philippines in the 1950’s with sporadic but infrequent outbreaks occurring over the next several decades [18,19,20]. Since 2011, an increasing number of chikungunya outbreaks have been reported to the Philippine Department of Health (DOH) [20]. However, no prospective longitudinal community-based cohort study has detected a meaningful number of confirmed CHIKV infections. In this first year of a two-year study, we sought to characterize the proportion of subclinical and symptomatic CHIKV infections. We were able to identify >12 total CHIKV infections per 100 person-years of surveillance and classify these as subclinical or symptomatic.

## Methods

### Ethics Statement

The study was approved by the Institutional Review Boards of Vicente Sotto Memorial Medical Center (Philippine DOH) in Cebu City, Philippines, and the Walter Reed Army Institute of Research. Written informed consent was obtained from subjects ≥18 years old and from parents of subjects <18 years old. Written assent was obtained from children ≥12 years old.

### Study Location and Population

The study was conducted in Cebu City, Philippines, an urban center of >800,000 residents located within the central Visayas region, one of three major island groups in the Philippines. All subjects were enrolled from the community of Punta Princesa, a dense urban area of 0.96 sq km with approximately 27,000 residents (Philippine National Statistics Office, 2010).

### Prospective Longitudinal Cohort

A prospective longitudinal community-based seroepidemiological cohort study of acute febrile illness was initiated in 2012 in Cebu City primarily to evaluate the incidence of influenza and dengue virus infections and secondarily to evaluate the incidence of other causes of acute febrile illness. From March-May 2012, subjects ≥6 months of age were recruited by door-to-door canvassing within the community. Approximately 200 subjects were targeted for enrollment in each of five age groups: 6 months-5 years, 6–15 years, 16–30 years, 31–50 years, and >50 years old. Since this was an exploratory study, the target size of the cohort was largely based on logistical considerations. Only one subject per household was enrolled. At enrollment, demographic and health questionnaires were administered and baseline blood samples collected. Subjects were instructed to report any fevers during the study period, and were monitored for acute febrile illnesses by weekly telephone calls or home visits. Any fever history within the prior seven days triggered an acute illness visit by study nurses who performed a clinical assessment that included a standardized symptoms questionnaire and acute blood collection. All acute blood samples were collected within seven days of illness onset. Follow-up visits to assess clinical status were conducted at 2, 5 and 8 days after the acute visit. Nasal and throat swabs were also collected at the acute, 2, 5 and 8 day visits but are not part of the current analysis. At 3 weeks, a convalescent visit was performed that included a clinical assessment and convalescent blood collection. Twelve months after enrollment (approximately March-June 2013), routine follow-up visits were conducted that included blood collections. All blood samples were processed into serum aliquots on the day of collection and stored at -70°C. These banked sera were available for further chikungunya testing. Acute serum samples were tested by reverse transcriptase polymerase chain reaction (RT-PCR) to detect CHIKV RNA [21,22]. Paired acute and 3-week convalescent serum samples were tested by an in-house CHIKV IgM/IgG enzyme-linked immunosorbent assay (ELISA) [23]. Paired enrollment and 12-month serum samples were tested by CHIKV plaque reduction neutralization test (PRNT) [23].

### Laboratory Assays

#### CHIKV RT-PCR

Viral RNA was extracted from 140 ul of subject serum using viral RNA extraction kit (Qiagen, Valencia, CA, USA). A two-step nested PCR was modified from Porter *et al*. and Laras *et al*. to amplify the capsid region of CHIKV [21,22].

#### CHIKV IgM/IgG ELISA

An in-house quantitative anti-CHIKV capture IgM/IgG ELISA was modified from Innis *et al*. and performed as previously described [24,25]. Subject serum at 1:100 dilution, anti-CHIKV K42 monoclonal antibody (provided by Dr. Alan Schmaljohn, U.S. Army Medical Research Institute of Infectious Diseases, Frederick, MD, USA) diluted at 1:1000, and sucrose acetone-extracted CHIKV antigen (50 HA units) were utilized in the assay. CHIKV ELISA IgM ≥40 units was considered positive.

#### CHIKV PRNT

PRNT was performed using the procedure of Russell *et al*. with modifications as previously described [23,26]. Four-fold serial dilutions of subject serum starting at 1:10 were incubated with attenuated strain 181/clone25 derived from a CHIKV Asian strain isolated from Thailand in 1962 [27]. Virus-serum admixtures were incubated on a monolayer of Macaca mulatta kidney (LLC-MK2) cells in a 12-well plate with 30–50 plaques in control wells. PRNT data was expressed as the reciprocal of the serum dilution causing 80% plaque reduction as extrapolated by probit regression.

#### CHIKV sequencing

Three CHIKV-positive acute blood samples were selected for E1 gene sequencing based on relatively strong CHIKV RT-PCR signal. Two additional CHIKV-positive acute blood samples were selected from different months of the year for viral isolation in *Ae*. *albopictus*-derived C6/36 cells as previously described [28], followed by sequencing of the isolate. DNA fragments of E1 gene were amplified using AccessQuick RT-PCR System (Promega, Madison, WI, USA) and purified using PCR purification kits (Qiagen, Valencia, CA, USA). Sanger sequencing of E1 gene was performed by AITbiotech (Singapore). Sequencher software (Gene Codes Corporation, Ann Arbor, MI, USA) was used to generate consensus sequences. E1 gene sequences of 1,320 bps (nucleotide position 999–11,313 of prototype CHIKV S27 gene sequence) were aligned with other CHIKV E1 sequences from GenBank to construct a phylogenetic tree using MEGA 6 software and neighbor-joining (maximum composite likelihood) methods with 1,000 replicates for bootstrap testing.

### Study Definitions

Acute symptomatic CHIKV infection was defined as a febrile illness with positive CHIKV PCR in the acute sample, or positive CHIKV IgM ELISA in the acute and/or convalescent samples with rising IgM levels, or ≥4-fold rise in CHIKV IgG ELISA in the paired acute/convalescent samples. Subclinical CHIKV infection was defined as ≥8-fold rise in the 12-month PRNT titer from a baseline enrollment titer of <10 with no acute symptomatic CHIKV infection detected during the intervening surveillance period. An enrollment PRNT titer ≥10 was considered to indicate past CHIKV infection.

### Statistical Analysis

Descriptive statistics for infection rates, symptoms and other characteristics were performed. Associations among categorical variables were estimated and tested for significance using chi-square or Fisher’s exact test as appropriate. P-value <0.05 was considered significant. Exact odds ratio (OR) and confidence interval (CI) for baseline PRNT status were calculated using conditional maximum likelihood estimates. Percent protection attributable to PRNT status was calculated as 100*(1-OR). All analyses were performed using R version 3.0.2 (R Foundation for Statistical Computing, Vienna, Austria).

## Results

**Fig 1:**
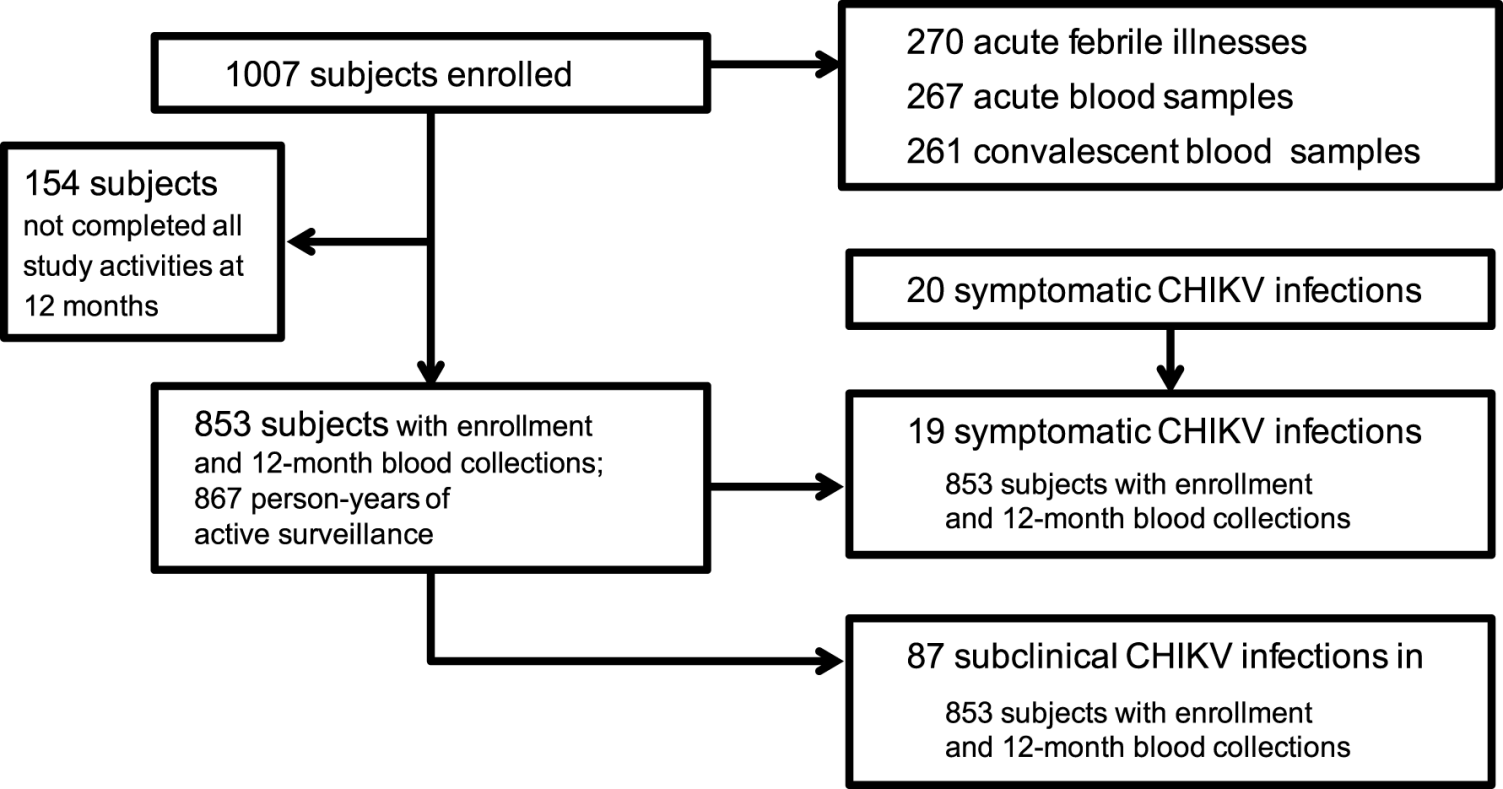
Study flow chart.

**Fig 2:**
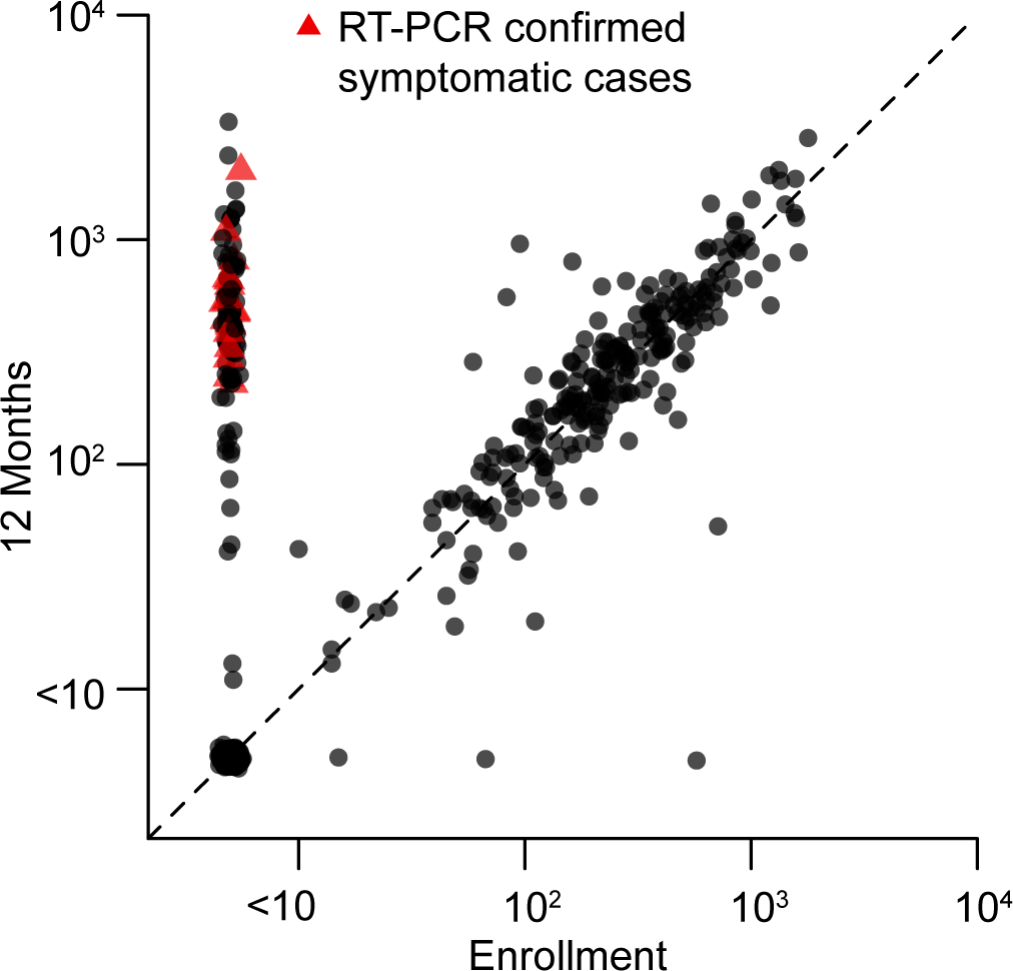
Dot plot of chikungunya virus (CHIKV) plaque reduction neutralization test (PRNT) titers at enrollment and 12 months in per-protocol subjects. All 19 symptomatic and 87 subclinical CHIKV infections had enrollment PRNT titer <10 and 12-month titers ranging from 229 to 2,030 in symptomatic infections and 64 to 3,347 in subclinical infections; 239 subjects had positive titers at enrollment ranging from 10 to 1,785; and 505 subjects had negative titers at both enrollment and 12 months.

**Fig 3:**
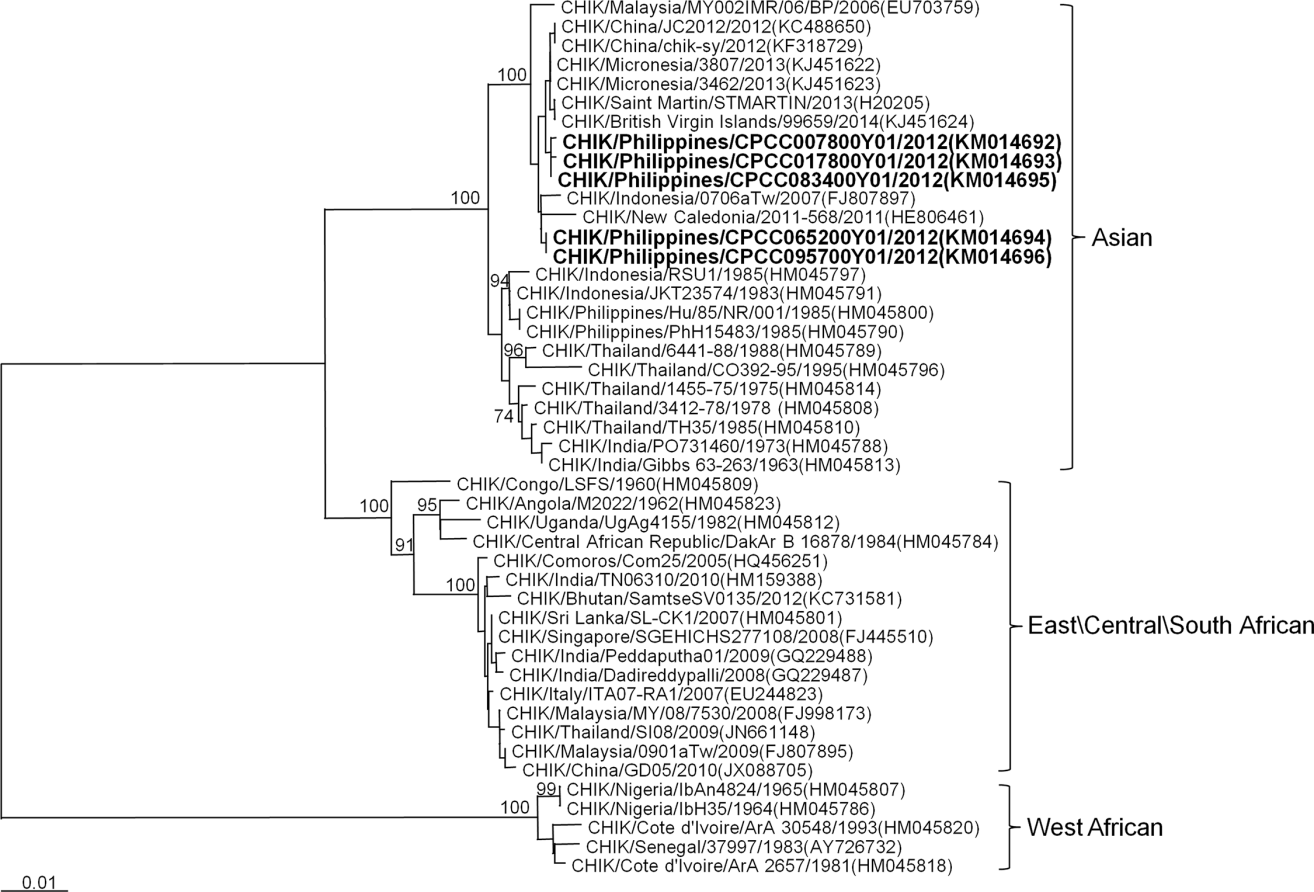
Phylogenetic tree showing five chikungunya viruses (CHIKVs) characterized in the study. The tree was constructed by neighbor-joining methods (1,000 bootstrap replications) using envelope protein-1 (E1) nucleotide sequences (1,320 bp) of 46 CHIKV strains; the five from the study are designated in bold. Bootstrap support values are shown for major nodes. Scale bar indicates nucleotide substitutions per site. Genotypes are indicated on the right. The sequences were named according to virus/country/strain/year of collection or isolation. GenBank accession numbers are shown in parentheses.

A total of 1007 subjects with approximately equal gender distribution were enrolled in the cohort with about 200 subjects contained in each of the five age groups (Table 1). Two hundred seventy (270) acute febrile illnesses were detected with 267 (98.9%) acute and 261 (96.7%) convalescent blood samples collected among 223 subjects. Twenty (7.5%) of 267 acute samples were CHIKV PCR-positive from 20 different individuals, of which all were also positive by ELISA. All subjects with ELISA seroconversion between acute and convalescent samples were also PCR-positive in the acute sample. Of the initial 1007 enrolled subjects, 853 completed all study activities per-protocol including enrollment and 12-month blood collections. Among these 853 per-protocol subjects, 19 symptomatic CHIKV infections occurred during 867 person-years of surveillance. An additional 87 subclinical CHIKV infections occurred in these per-protocol subjects based on ≥8-fold rise in 12-month PRNT titer from a baseline enrollment titer of <10 (Fig 1) [Note: PRNT values using 50% plaque reduction in addition to 80% were also calculated for all PRNT results. Two subjects meeting the definition of “subclinical infection” by PRNT80 values but with baseline enrollment PRNT50 titer of ≥10 were not included among the 87 subclinical infections due to the possibility of prior CHIKV infection.]. The 12-month PRNT titers ranged from 229 to 2,030 in symptomatic infections and from 64 to 3,347 in subclinical infections (Fig 2). The incidence of symptomatic CHIKV infection per-protocol, therefore, was 2.19 per 100 person-years (95%CI: 1.36, 3.35), and of subclinical infection per-protocol was 10.03 per 100 person-years (95%CI: 8.09, 12.31). Sequencing of the E1 gene from five PCR-positive samples/isolates revealed all five to be Asian genotype (GenBank accession numbers KM014692 to KM014696), closely related to strains from New Caledonia (2011), China (2012), Micronesia/Yap (2013), Saint Martin (2013) and British Virgin Islands (2014) (Fig 3).

**Table 1:** Description of cohort subjects.

Characteristic	n (%)
Enrolled subjects	1007 (100)
Per-protocol subjects*	853 (84.7)
Age: Enrolled subjects / Per-protocol subjects	
6 mos—5 yrs	203 (20.2)/ 148 (17.4)
6–15 yrs	201 (20.0) / 184 (21.6)
16–30 yrs	200 (19.9) / 168 (19.7)
31–50 yrs	202 (20.1) / 170 (19.9)
>50 yrs	201 (20.0) / 183 (21.5)
All ages	1007 (100) / 853 (100)
Male gender at enrollment	501 (49.8)
Number in household at enrollment	
1	16 (1.6)
2–3	206 (20.5)
4–6	528 (52.4)
7–10	235 (23.3)
>10	22 (2.2)
Number of children in household at enrollment	
0	179 (17.8)
1	224 (22.2)
2	228 (22.6)
3	183 (18.2)
>3	193 (19.2)

*Subjects who completed all study activities at 12 months including enrollment and 12-month blood collections.

n = number.

The incidence of symptomatic CHIKV infection in different age groups ranged from 0.55 (>50 years old) to 4.23 (6–15 years old) per 100 person-years (see Table 2 for rates in each age group with confidence intervals). The incidence of subclinical CHIKV infection ranged from 6.46 (6 months-5 years old) to 12.68 (6–15 years old) per 100 person-years (Table 2). The ratio of subclinical-to-symptomatic infections ranged from 2:1 (6 months-5 years old) to 12:1 (>50 years old). Seroprevalence at enrollment of positive CHIKV PRNT titer (i.e., ≥10) increased with each older age group although the degree of increase with each age group varied (Table 2). Among just those subjects with negative CHIKV PRNT at enrollment, the incidence of symptomatic CHIKV infection ranged from 1.41 (>50 years old) to 4.27 (6–15 years old) per 100 person-years; subclinical CHIKV infection ranged from 6.51 (6 months-5 years old) to 26.7 (31–50 years old) per 100 person-years; total CHIKV infection ranged from 9.77 (6 months-5 years old) to 30.51 (31–50 years old) per 100 person-years (see Table 3 for rates in each age group with confidence intervals). The ratio of subclinical-to-symptomatic infections was the same as in all per-protocol subjects since all CHIKV infections occurred in subjects with negative baseline PRNT titer.

**Table 2:** Incidence of CHIKV infections and seroprevalence of CHIKV PRNT in different age groups.^a^

Age	Subjects, n	CHIKV PRNT ≥10, n (% of age group)^b^	Symptomatic CHIKV infection, n [n/100 person-yrs (95% CI)]	Subclinical CHIKV infection, n [n/100 person-yrs (95% CI)]	Total CHIKV infection, n [n/100 person-yrs (95% CI)]	Ratio of subclinical to symptomatic CHIKV infection
6 mos—5 yrs	148	1 (0.7)	5 [3.23 (1.23, 7.08)]	10 [6.46 (3.32, 11.47)]	15 [9.69 (5.66, 15.59)]	2:1
6–15 yrs	184	2 (1.1)	8 [4.23 (1.99, 7.98)]	24 [12.68 (8.33, 18.55)]	32 [16.91 (11.78, 23.56)]	3:1
16–30 yrs	168	34 (20.2)	2 [1.13 (0.23, 3.63)]	20 [11.32 (7.13, 17.14)]	22 [12.45 (8.02, 18.51)]	10:1
31–50 yrs	170	90 (52.9)	3 [1.8 (0.50, 4.81)]	21 [12.63 (8.05, 18.94)]	24 [14.43 (9.49, 21.12)]	7:1
>50 yrs	183	112 (61.2)	1 [0.55 (0.05, 2.59)]	12 [6.66 (3.64, 11.28)]	13 [7.21 (4.04, 11.98)]	12:1
All ages	853	239 (28.0)	19 [2.19 (1.36, 3.35)]	87 [10.03 (8.09, 12.31)]	106 [12.22 (10.06, 14.72)]	4.6:1

^a^Based on 853 subjects with both enrollment and 12-month blood collections.

^b^CHIKV PRNT titer at enrollment using 80% plaque reduction.

CHIKV = chikungunya virus; n = number; CI = confidence interval; PRNT = plaque reduction neutralization test.

**Table 3:** Incidence of CHIKV infections among individuals with negative baseline CHIKV PRNT in different age groups.^a^

Age	Subjects with negative CHIKV PRNT^b^, n	Symptomatic CHIKV infection, n [n/100 person-yrs (95% CI)]	Subclinical CHIKV infection, n [n/100 person-yrs (95% CI)]	Total CHIKV infection, n [n/100 person-yrs (95% CI)]
6 mos—5 yrs	147	5 [3.26 (1.23, 7.14)]	10 [6.51 (3.34, 11.55)]	15 [9.77 (5.71, 15.70)]
6–15 yrs	182	8 [4.27 (2.02, 8.06)]	24 [12.82 (8.43, 18.76)]	32 [17.09 (11.91, 23.82)]
16–30 yrs	134	2 [1.42 (0.28, 4.56)]	20 [14.22 (8.96, 21.52)]	22 [15.64 (10.08, 23.25)]
31–50 yrs	80	3 [3.81 (1.06, 10.18)]	21 [26.70 (17.02, 40.04)]	24 [30.51 (20.05, 44.64)]
>50 yrs	71	1 [1.41 (0.13, 6.60)]	12 [16.98 (9.27, 28.75)]	13 [18.39 (10.30, 30.55)]
All ages	614	19 [3.01 (1.87, 4.61)]	87 [13.79 (11.12, 16.92)]	106 [16.80 (13.83, 20.24)]

^a^Based on 614 subjects with both enrollment and 12-month blood collections who had negative baseline CHIKV PRNT titer.

^b^CHIKV PRNT titer at enrollment using 80% plaque reduction.

CHIKV = chikungunya virus; n = number; CI = confidence interval; PRNT = plaque reduction neutralization test.

Clinical features of all 20 symptomatic CHIKV infections (of which 19 occurred in per-protocol subjects) are shown in Table 4. Only two (10%) subjects sought medical care; one was hospitalized. Arthralgia was present in nine (45%) cases: 3/14 (21%) subjects ≤15 years old and 6/6 (100%) subjects >15 years old. At the 3-week convalescent visit, symptoms had completely resolved in 18 (90%) subjects. The remaining two subjects had rhinorrhea/nasal congestion and/or cough at 3 weeks.

**Table 4:** Clinical presentation of symptomatic CHIKV infections.^a^

Clinical parameter	6 mos—5 yrs, n (%)	6–15 yrs, n (%)	16–30 yrs, n (%)	31–50 yrs, n (%)	>50 yrs, n (%)	All ages, n (%)
Fever history	6 (100)	8 (100)	2 (100)	3 (100)	1 (100)	20 (100)
Headache	4 (66.6)	7 (87.5)	2 (100)	1 (33.3)	1 (100)	15 (75)
Rash	5 (83.3)	6 (75)	2 (100)	1 (33.3)	1 (100)	15 (75)
Anorexia	3 (50)	3 (37.5)	2 (100)	3 (100)	1 (100)	12 (60)
Myalgia	2 (33.3)	4 (50)	2 (100)	3 (100)	1 (100)	12 (60)
Arthralgia	2 (33.3)	1 (12.5)	2 (100)	3 (100)	1(100)	9 (45)
Chills	2 (33.3)	2 (25)	2 (100)	2 (66.7)	1 (100)	9 (45)
Conjunctival irritation	2 (33.3)	4 (50)	1 (50)	2 (66.7)	0 (0)	9 (45)
Rhinorrhea/nasal congestion	3 (50)	3 (37.5)	1 (50)	1 (33.3)	0 (0)	8 (40)
Cough	2 (33.3)	2 (25)	2 (100)	1 (33.3)	1 (100)	8 (40)
Abdominal pain	2 (33.3)	3 (37.5)	2 (100)	0 (0)	0 (0)	7 (35)
Sore throat	0 (0)	2 (25)	1 (50)	2 (66.7)	1 (100)	6 (30)
Nausea/vomiting	2 (33.3)	1 (12.5)	0 (0)	2 (66.7)	0 (0)	5 (25)
Hoarseness	1 (16.7)	0 (0)	0 (0)	1 (33.3)	1 (100)	3 (15)
Diarrhea	1 (16.7)	0 (0)	0(0)	0 (0)	0(0)	1 (5)
Sought outpatient medical care	1 (16.7)	0 (0)	0 (0)	0 (0)	0 (0)	1 (5)
Hospitalized	0 (0)	1 (12.5)	0 (0)	0 (0)	0 (0)	1 (5)

^a^Based on 20 symptomatic CHIKV infections and on clinical parameters present at any one of the acute, 2, 5, or 8 day visits.

CHIKV = chikungunya virus; n = number.

The relationship between occurrence of symptomatic CHIKV infection and baseline enrollment CHIKV PRNT titer is shown in Table 5. PRNT data was available only in per-protocol subjects. Among the 853 per-protocol subjects, all 19 symptomatic CHIKV infections occurred in subjects with baseline PRNT titer <10 (Table 5). A positive baseline CHIKV PRNT titer (i.e., ≥10) was associated with 100% (95%CI: 46.1, 100.0) protection from symptomatic CHIKV infection. A positive baseline PRNT titer was present in 239 per-protocol subjects with titers ranging from 10 to 1,785 (Fig 2).

**Table 5:** Relationship between symptomatic CHIKV infection and baseline enrollment CHIKV PRNT status.^a^

Baseline CHIKV PRNT titer^b^	Symptomatic CHIKV infection present, n (%)	Symptomatic CHIKV infection absent, n (%)	Total, n (%)	% Protection when CHIKV PRNT ≥10 (95% CI)	P-value
<10	19 (3.1)	595 (96.9)	614 (100)	100.0 (46.1, 100.0)	0.003
≥10	0 (0)	239 (100)	239 (100)		

^a^Based on 853 subjects with both enrollment and 12-month blood collections.

^b^CHIKV PRNT titer at enrollment using 80% plaque reduction of attenuated vaccine strain 181/clone25.

CHIKV = chikungunya virus; PRNT = plaque reduction neutralization test; n = number; CI = confidence interval.

## Discussion

The incidence of CHIKV infection in our study conducted in a non-naïve population, where CHIKV has been endemic for decades, was relatively high with the ratio of subclinical-to-symptomatic infections notably greater than previous estimates [6,11,12,13,14,17]. The incidence and relative proportion of subclinical infection appeared to be age-dependent. This is one of the first prospective longitudinal seroepidemiological cohorts undergoing active surveillance with sufficient chikungunya incidence to characterize the proportion of subclinical and symptomatic CHIKV infections in an endemic region. In so doing, our results support CHIKV PRNT titer as a potential immune correlate of protection from infection.

The proportion of subclinical CHIKV infections in our study was 82.0% compared to 3.8–27.7% in prior studies [6]. This difference could be due to our study design in which baseline blood samples were collected in a defined community-based cohort prior to infection followed by the implementation of a sensitive active surveillance system to detect infection. Thus, we were able to characterize subclinical versus symptomatic infections more accurately than past seroprevalence studies which relied on obtaining retrospective histories of symptoms consistent with chikungunya [11,12,13,14]. Of note, since the active surveillance in our study focused on detecting febrile illness, it is possible that some subjects with “subclinical” infection may have had no fevers but still had non-febrile symptoms. For example, arthralgia without fever has been documented in a small percentage of acute CHIKV infections in prior studies [29,30]. Additionally, the genotype or strain of the infecting virus may have been a factor in the high proportion of subclinical infections. Most studies that have estimated the rate of subclinical infections have been conducted during ECSA genotype outbreaks whereas our study involved Asian genotype [11,12,13,14]. Interestingly, a large study of chikungunya caused by Asian genotype in 1962 in Bangkok, Thailand, estimated that 25% of Bangkok children had experienced CHIKV infection based on hemagglutination inhibition (HAI) seroconversion between the start and end of rainy season while up to 7% of all Bangkok children were estimated to have sought outpatient medical care for chikungunya, implying the majority of infected children had subclinical or mild infection [31]. A recent small study of blood donors from the Caribbean island of Saint Martin in 2014 estimated a substantial asymptomatic rate of 40% [32]. Clearly, the relevance of genotype or strain to clinical presentation requires further investigation. Finally, even the symptomatic CHIKV infections in our study were clinically mild with only two subjects seeking medical care, and all with complete resolution or minimal symptoms by 3 weeks. The overall high rate of both subclinical and mild infections suggests that the more clinically-apparent chikungunya cases typically reported to public health authorities may represent a small fraction of all infections. This has important implications for estimating disease burden, understanding virus transmission, and assessing blood transfusion risk. The role of subclinical and mild CHIKV infections in transmitting virus is incompletely understood. However, two blood donors with asymptomatic infections from the ongoing Caribbean outbreak have been shown to harbor viable CHIKV [33]. This finding suggests that at least some of the subclinical infections in our study could potentially participate in virus transmission.

Although CHIKV infections occurred in all age groups, the incidence of total and subclinical infections seemed to vary with age. Considering only subjects with negative CHIKV PRNT titer at enrollment (among whom all infections occurred), the incidence of total infections was lowest in subjects 6 months-5 years old. Moreover, the ratio of subclinical-to-symptomatic infection was higher in individuals >15 years old than in those between 6 months and 15 years old, although the ratio in all ages was quite high. Whether these differences represent typical age-related patterns or were specific to our study setting is unclear. Many factors could contribute to these age differences including age-related behavior patterns possibly leading to higher vector exposure in adolescents and adults, higher mosquito feeding intensity in adults compared to infants and toddlers [34], and more extensive immune histories in adults than children not necessarily reflected by PRNT titers that may have contributed to disease prevention or mitigation. For example, some older adults with negative baseline PRNT titers may, in fact, have had past infection such that subsequent CHIKV exposure caused a boost in PRNT titer. This situation would be less likely in children and in naïve populations. Thus, in countries where CHIKV has not previously circulated, the proportion of mild and subclinical infections may be lower than in our study. It is also possible that some of the differences among age groups may have been an artifact of relatively low case numbers in some groups.

The relatively high incidence of CHIKV infection in our study occurred in the Philippines where sporadic outbreaks have been reported for several decades but with increasing frequency over the past three years. Cebu province has never reported a chikungunya outbreak and has documented very few individual cases including during the current study period (communication, Dr. Vito G. Roque, Philippine DOH). The nonspecific nature of symptoms and the high rate of subclinical and mild infections may partly account for this lack of reported cases. Additional prospective cohort studies with active fever surveillance may be highly informative even in regions with few reported cases.

Our study allowed for the evaluation of potential immune correlates of protection from CHIKV infection in humans. Previous animal studies have demonstrated that CHIKV neutralizing antibodies are associated with protection in mice [35,36] and non-human primates [37,38]. Human studies of CHIKV antibodies following infection have suggested neutralizing antibodies correlate with viral clearance and long-term clinical protection [39,40]. Our results demonstrated that a positive baseline CHIKV PRNT titer was associated with protection from symptomatic CHIKV infection in humans. This is one of the first human field studies to find such an association and will contribute to the increasing body of evidence supporting CHIKV neutralizing antibodies as an immune correlate of protection from infection.

Although not included among those with symptomatic and subclinical CHIKV infections, four subjects with an enrollment PRNT titer ≥10 had a 4 to 8-fold rise in PRNT titer at 12 months (increasing from 10 to 42, 59 to 286, 83 to 555, and 162 to 799, respectively), and one subject had a ≥8-fold rise (from 95 to 959). It is unclear whether these results were due to assay variability or to immunological boosting after virus exposure. The ≥8-fold rise was probably due to the latter. In any event, the small number of such cases and the relatively small increase in titer suggest that most subjects with a positive PRNT titer probably had a certain degree of sterile immunity.

Several limitations of the study should be noted. First, the results are from a single study year in a limited study population and geographic area potentially limiting the generalizability of the results. Second, the CHIKV strain identified in this study was Asian genotype which could behave differently from other CHIKV genotypes such as ECSA. Nevertheless, the findings are directly applicable to closely-related Asian strains such as those detected recently from the Caribbean outbreak. Third, although serological cross-reactivity with other alphaviruses is theoretically possible, the relatively large number of CHIKV PCR-positive samples in our study combined with the extremely low CHIKV seroprevalence in subjects ≤15 years old suggesting minimal CHIKV activity over the past 15 years, and the lack of any confirmed non-CHIKV human alphavirus infections in the Philippines make it unlikely that other alphaviruses were responsible for the seropositivity seen in our study. Fourth, although active surveillance was used to identify febrile episodes, some illnesses may have gone undetected leading to an overestimation of subclinical infections. Finally, the relatively low number of symptomatic infections led to relatively large confidence intervals. Therefore, a larger number of CHIKV infections in future studies may be necessary to confirm our findings.

In summary, subclinical and mild CHIKV infections may account for a much larger proportion of total infections than previously reported, with substantial underreporting to public health systems. This has implications for accurate assessments of chikungunya disease burden, virus transmission, and blood transfusion risk. The finding that a positive CHIKV PRNT titer was associated with protection from infection will contribute to ongoing vaccine development efforts. Additional prospective longitudinal cohort studies should be done to validate our findings in other regions with known chikungunya activity.

## Supporting Information

S1 Checklist STROBE checklist. (DOCX)

S1 Questionnaire Symptoms questionnaire. List of symptoms asked by study staff during acute febrile episode investigations.
(DOCX)
