# Supplementary material for: High Rate of Subclinical Chikungunya Virus Infection and Association of Neutralizing Antibody with Protection in a Prospective Cohort in The Philippines
Source: PLoS Negl Trop Dis. 2015 May 7;9(5):e0003764. doi: 10.1371/journal.pntd.0003764 (PMC4423927; doi:10.1371/journal.pntd.0003764)
Supplement: S1 Checklist — (DOCX) [file pntd.0003764.s001.docx]

STROBE Statement—Checklist of items that should be included in reports of ***cohort studies***

|  | Item No | Recommendation |
| --- | --- | --- |
| **Title and abstract** | 1 | (*a*) Indicate the study’s design with a commonly used term in the title or the abstract  **The title states “prospective cohort.”** |
|  |  | (*b*) Provide in the abstract an informative and balanced summary of what was done and what was found  **This was provided in the abstract.** |
| Introduction | | |
| Background/rationale | 2 | Explain the scientific background and rationale for the investigation being reported  **The introduction states: “It has been presumed that a majority of infections present with acute fever accompanied by other overt symptoms. However, assumptions about the clinical presentation of CHIKV infection have largely been based on sentinel surveillance and cross-sectional serosurveys.”** |
| Objectives | 3 | State specific objectives, including any prespecified hypotheses  **The introduction states: “we sought to characterize the full clinical spectrum of CHIKV infection.”** |
| Methods | | |
| Study design | 4 | Present key elements of study design early in the paper  **This was presented early in the Methods section under the sub-section “Prospective Longitudinal Cohort.”** |
| Setting | 5 | Describe the setting, locations, and relevant dates, including periods of recruitment, exposure, follow-up, and data collection  **This was described in the Methods section under the sub-sections “Study Location and Population” and “Prospective Longitudinal Cohort.”** |
| Participants | 6 | (*a*) Give the eligibility criteria, and the sources and methods of selection of participants. Describe methods of follow-up.  **This was described in the Methods section under the sub-section “Prospective Longitudinal Cohort.” Convenience sampling through door-to-door canvassing within a defined urban community was used to recruit subjects.** |
|  |  | (*b*) For matched studies, give matching criteria and number of exposed and unexposed  **Not applicable.** |
| Variables | 7 | Clearly define all outcomes, exposures, predictors, potential confounders, and effect modifiers. Give diagnostic criteria, if applicable  **This was described in the Methods section under the sub-section “Prospective Longitudinal Cohort.” Laboratory definitions were described in the Methods section under the sub-section “Laboratory Assays.” Symptomatic CHIKV infections were defined as illnesses with reported history of fever with specific laboratory criteria. Subclinical infections were defined as those without reported history of fever but with certain laboratory criteria.** |
| Data sources/ measurement | 8* | For each variable of interest, give sources of data and details of methods of assessment (measurement). Describe comparability of assessment methods if there is more than one group  **This was described in the Methods section under the sub-section “Prospective Longitudinal Cohort.” Laboratory definitions were described in the Methods section under the sub-section “Laboratory Assays.”** |
| Bias | 9 | Describe any efforts to address potential sources of bias  **This was described in the Methods section under the sub-section “Prospective Longitudinal Cohort.” A roughly equal number of subjects were targeted for recruitment from each age group. Only one subject was recruited from each household.** |
| Study size | 10 | Explain how the study size was arrived at  **The Methods section under the sub-section “Prospective Longitudinal Cohort” states: “Since this was an exploratory study, the target size of the cohort was largely based on logistical considerations.”** |
| Quantitative variables | 11 | Explain how quantitative variables were handled in the analyses. If applicable, describe which groupings were chosen and why  **Age groups for enrolment were determined prior to study initiation based on commonly used age categories. Since this was an exploratory study, the age groupings were arbitrary. Laboratory definitions were described in the Methods section under the sub-section “Laboratory Assays.” Cut-off value for positive versus negative neutralizing antibody titers was based on the standard initial dilution for the assay.** |
| Statistical methods | 12 | (*a*) Describe all statistical methods, including those used to control for confounding  **Basic statistical methods were used for analysis as summarized in the “Statistical Methods” sub-section. Statistical methods specifically targeting confounders were not performed.** |
|  |  | (*b*) Describe any methods used to examine subgroups and interactions  **Basic statistical methods were used for analysis as summarized in the “Statistical Methods” sub-section. Statistical methods specifically targeting interactions were not performed.** |
|  |  | (*c*) Explain how missing data were addressed  **Any relevant missing data were listed in the paper. These were minimal and were not included in any analyses.** |
|  |  | (*d*) If applicable, explain how loss to follow-up was addressed  **Subjects who completed all protocol activities were designated as “per-protocol” subjects. Most of the analyses such as determination of subclinical infection rates and neutralizing antibody correlation with protection could only be done on “per protocol” subjects who completed all study activities.** |
|  |  | (*e*) Describe any sensitivity analyses  **Not performed.** |
| Results | | |
| Participants | 13* | (a) Report numbers of individuals at each stage of study—eg numbers potentially eligible, examined for eligibility, confirmed eligible, included in the study, completing follow-up, and analysed  **These were listed in the Results section and in Figure 1.** |
|  |  | (b) Give reasons for non-participation at each stage  **Most of the analyses could only be done on “per-protocol” subjects who completed all study activities. However, compliance with the protocol procedures was generally very good.** |
|  |  | (c) Consider use of a flow diagram  **See Figure 1.** |
| Descriptive data | 14* | (a) Give characteristics of study participants (eg demographic, clinical, social) and information on exposures and potential confounders  **See Table 1.** |
|  |  | (b) Indicate number of participants with missing data for each variable of interest  **There was no relevant missing data. Most of the basic analyses could only be done on “per-protocol” subjects who completed all study activities.** |
|  |  | (c) Summarise follow-up time (eg, average and total amount)  **This is summarized throughout the paper as person-years of surveillance. In addition, much of the analyses could only be performed on “per-protocol” subjects.** |
| Outcome data | 15* | Report numbers of outcome events or summary measures over time  **This is reported in the Results section.** |
| Main results | 16 | (*a*) Give unadjusted estimates and, if applicable, confounder-adjusted estimates and their precision (eg, 95% confidence interval). Make clear which confounders were adjusted for and why they were included  **This is provided in the Results section.** |
|  |  | (*b*) Report category boundaries when continuous variables were categorized  **Neutralizing antibody titers were categorized as negative (<10) or positive (≥10).** |
|  |  | (*c*) If relevant, consider translating estimates of relative risk into absolute risk for a meaningful time period  **Not applicable.** |
| Other analyses | 17 | Report other analyses done—eg analyses of subgroups and interactions, and sensitivity analyses  **Relevant further analyses are presented in the Results section.** |
| Discussion | | |
| Key results | 18 | Summarise key results with reference to study objectives  **Symptomatic and subclinical CHIKV infection rates are summarized in the Discussion section.** |
| Limitations | 19 | Discuss limitations of the study, taking into account sources of potential bias or imprecision. Discuss both direction and magnitude of any potential bias  **Relevant study limitations are described in a separate paragraph in the Discussion section.** |
| Interpretation | 20 | Give a cautious overall interpretation of results considering objectives, limitations, multiplicity of analyses, results from similar studies, and other relevant evidence  **This is provided in the Discussion section.** |
| Generalisability | 21 | Discuss the generalisability (external validity) of the study results  **This is mentioned in the Discussion section in that the study results are most relevant to the current chikungunya epidemic in the Americas.** |
| Other information | | |
| Funding | 22 | Give the source of funding and the role of the funders for the present study and, if applicable, for the original study on which the present article is based  **The funding sources were mentioned.** |

*Give information separately for exposed and unexposed groups.

**Note:** An Explanation and Elaboration article discusses each checklist item and gives methodological background and published examples of transparent reporting. The STROBE checklist is best used in conjunction with this article (freely available on the Web sites of PLoS Medicine at http://www.plosmedicine.org/, Annals of Internal Medicine at http://www.annals.org/, and Epidemiology at http://www.epidem.com/). Information on the STROBE Initiative is available at http://www.strobe-statement.org.
