# Supplementary material for: High Rate of Subclinical Chikungunya Virus Infection and Association of Neutralizing Antibody with Protection in a Prospective Cohort in The Philippines
Source: PLoS Negl Trop Dis. 2015 May 7;9(5):e0003764. doi: 10.1371/journal.pntd.0003764 (PMC4423927; doi:10.1371/journal.pntd.0003764)
Supplement: S1 Questionnaire — List of symptoms asked by study staff during acute febrile episode investigations. (DOCX) [file pntd.0003764.s002.docx]

**THIS SCHEDULE SHOULD ONLY BE COMPLETED FOR COHORT PARTICIPANTS WITH FEVER OR HISTORY OF FEVER (DEFINED AS REPORTED FEVER WITHIN 7 DAYS OF PRESENTATION OR MEASURED TEMPERATURE OF ≥ 38^O^C).**

E3. Interview Date _______ / _______ / ______________

Day Month Year

E4. Follow-up Week # _____ _____ E5. Event # _____ _____

**FEVER HISTORY**

I will begin by asking questions about your fever this past week.

E6. What date did you begin having symptoms such as fever, cough or sore throat?

_______ / _______ / ______________

Day Month Year

E7. In the past 7 days, have you had any fever?

Yes 1 *IF YES THEN GO TO E8*

No 2 *IF NO THEN READ BOX PG 2 AND GO TO E13,*

*TABLE E1*

Don’t Know 7 *IF DK THEN READ BOX PG 2 AND GO TO E13,*

*TABLE E1*

Refused 8 *IF RF THEN READ BOX PG 2 AND GO TO E13,*

*TABLE E1*

E8. What is the highest temperature you measured while having a fever in the past 7 days?

_____ _____ . _____ ^o^C

Don’t Know 77

Refused 88

E9. How did you measure your temperature? Was it...

By mouth 1 By touch 5

From the ear 2 Don’t Know 7

Under the arm 3 Refused 8

Rectally 4

E10. How many days did your fever last?

_____ _____ days

Don’t Know 77

Refused 88

E11. In the past week, have you been absent from work/ school/ day care because of fever?

Yes 1 *IF YES THEN GO TO E12*

No 2 *IF NO THEN READ BOX BELOW AND GO TO E13, TABLE E1*

Don’t Know 7 *IF DK THEN READ BOX BELOW AND GO TO E13, TABLE E1*

Refused 8 *IF RF THEN READ BOX BELOW AND GO TO E13, TABLE E1*

E12. How many days of work/ school /day care did you miss due to your fever?

_____ _____ days *ENTER 0 FOR LESS THAN 1 DAY*

Don’t Know 7

Refused 8

Now, I am going to read a list of symptoms to you. Please tell me yes or no if you have had any of these symptoms since your sickness began.

***COMPLETE TABLE E1: SYMPTOMS***

| TABLE E1: SYMPTOMS | | | | | | | | | | |
| --- | --- | --- | --- | --- | --- | --- | --- | --- | --- | --- |
| Q# | Symptom | YesGO TO NEXT Q# | NoGO TO NEXT CONDITION | DK | REF | Q# | Treated at hospital? | | Q# | Hospital or Clinic Name |
|  |  |  |  |  |  |  | YesGO TO NEXT Q# | NoGO TO NEXT CONDITION |  |  |
| E13 | Headache | 1 | 2 | 7 | 8 | E14 | 1 | 2 | E15 |  |
| E16 | Red or itchy or watery eyes | 1 | 2 | 7 | 8 | E17 | 1 | 2 | E18 |  |
| E19 | Runny or stuffy nose | 1 | 2 | 7 | 8 | E20 | 1 | 2 | E21 |  |
| E22 | Sore throat | 1 | 2 | 7 | 8 | E23 | 1 | 2 | E24 |  |
| E25 | Hoarseness | 1 | 2 | 7 | 8 | E26 | 1 | 2 | E27 |  |
| E28 | Cough | 1 | 2 | 7 | 8 | E29 | 1 | 2 | E30 |  |
| E31 | Shortness of breath | 1 | 2 | 7 | 8 | E32 | 1 | 2 | E33 |  |
| E34 | Nausea or vomiting | 1 | 2 | 7 | 8 | E35 | 1 | 2 | E36 |  |
| E37 | Stomach pain | 1 | 2 | 7 | 8 | E38 | 1 | 2 | E39 |  |
| E40 | Diarrhea  (having more than 3 loose bowl movements in a 24 hour period) | 1 | 2 | 7 | 8 | E41 | 1 | 2 | E42 |  |
| E43 | Rash | 1 | 2 | 7 | 8 | E44 | 1 | 2 | E45 |  |
| E46 | Anorexia (loss or appetite, not wanting to eat) | 1 | 2 | 7 | 8 | E47 | 1 | 2 | E48 |  |
| E49 | Chills | 1 | 2 | 7 | 8 | E50 | 1 | 2 | E51 |  |
| E52 | Muscle aches | 1 | 2 | 7 | 8 | E53 | 1 | 2 | E54 |  |
| E55 | Joint pain | 1 | 2 | 7 | 8 | E56 | 1 | 2 | E57 |  |
| E58 | Other, describe: | 1 | 2 | 7 | 8 | E59 | 1 | 2 | E60 |  |

E61. Have you had any bleeding since your illness began?

Yes 1 *IF YES THEN GO TO E62*

No 2 *IF NO THEN GO TO E68*

Don’t Know 7 *IF DK THEN GO TO E60*

Refused 8 *IF REFUSED THEN GO TO E60*

From what part of your body have you been bleeding? I will read a list of possible

sites. Please tell me yes or no if you have had bleeding from any of these parts of your body since your illness began.

| TABLE E2: BLEEDING SITES |
| --- |

| **Q#** | Site | Yes | No | Don’t Know | Refused |
| --- | --- | --- | --- | --- | --- |
| E62 | Nose | 1 | 2 | 7 | 8 |
| E63 | Gums | 1 | 2 | 7 | 8 |
| E64 | Eyes | 1 | 2 | 7 | 8 |
| E65 | Ear | 1 | 2 | 7 | 8 |
| E66 | Stomach | 1 | 2 | 7 | 8 |
| E67 | Skin | 1 | 2 | 7 | 8 |
| E68 | Vomiting blood | 1 | 2 | 7 | 8 |
| E69 | Blood in urine | 1 | 2 | 7 | 8 |
| E70 | Blood in stool | 1 | 2 | 7 | 8 |
